# Effect of Process Parameters and Build Orientation on Microstructure and Impact Energy of Electron Beam Powder Bed Fused Ti-6Al-4V

**DOI:** 10.3390/ma14185376

**Published:** 2021-09-17

**Authors:** Spencer Jeffs, Robert Lancaster, Gareth Davies, William Hole, Brenna Roberts, David Stapleton, Meurig Thomas, Iain Todd, Gavin Baxter

**Affiliations:** 1 Bay Campus, Institute of Structural Materials, Faculty of Science and Engineering, Swansea University, Swansea SA1 8EN, UK; r.j.lancaster@swansea.ac.uk (R.L.); 958316@Swansea.ac.uk (W.H.); 904837@Swansea.ac.uk (B.R.); 2 Rolls-Royce plc, P.O. Box 31, Derby DE24 8BJ, UK; Gareth.Davies9@Rolls-Royce.com (G.D.); david.stapleton2@rolls-royce.com (D.S.); 3 Department of Materials Science and Engineering, University of Sheffield, Sir Robert Hadfield Building, Mappin Street, Sheffield S1 3JD, UK; meurig.thomas@sheffield.ac.uk (M.T.); iain.todd@sheffield.ac.uk (I.T.); g.j.baxter@sheffield.ac.uk (G.B.)

**Keywords:** additive manufacturing, electron beam powder bed fusion, impact testing

## Abstract

To fully exploit the benefits of additive manufacturing (AM), an understanding of its processing, microstructural, and mechanical aspects, and their interdependent characteristics, is necessary. In certain instances, AM materials may be desired for applications where impact toughness is a key property, such as in gas turbine fan blades, where foreign or direct object damage may occur. In this research, the impact energy of a series of Ti-6Al-4V specimens produced via electron beam powder bed fusion (EBPBF) was established via Charpy impact testing. Specimens were produced with five different processing parameter sets, in both the vertical and horizontal build orientation, with microstructural characteristics of prior β grain area, prior β grain width, and α lath width determined in the build direction. The results reveal that horizontally oriented specimens have a lower impact energy compared to those built in the vertical orientation, due to the influence of epitaxial grain growth in the build direction. Relationships between process parameters, microstructural characteristics, and impact energy results were evaluated, with beam velocity displaying the strongest trend in terms of impact energy results, and normalised energy density exhibiting the most significant influence across all microstructural measurements.

## 1. Introduction

Additive manufacturing (AM) is a layer-by-layer manufacturing technique that builds up components from a common feedstock such as powder or wire. Metal AM has seen significant interest from multiple industries due to its ability to manufacture complex and intricate components with a high degree of design freedom that may not be possible with subtractive machining techniques. Significant review articles have been published on metal AM that provide a comprehensive overview of the topic [1,2]. Electron beam powder bed fusion (EBPBF) is a prominent AM method that uses an electron beam as the energy source to melt successive powder layers, typically under vacuum conditions. Key process variables of EBPBF include beam power, beam velocity, hatch spacing, and layer height. EBPBF has been employed across several material systems, including titanium alloys, nickel-based superalloys, and tungsten alloys [3,4,5].

Ti-6Al-4V is an α + β titanium alloy with a good balance of properties, such as specific strength, corrosion resistance, and biocompatibility, which is used in multiple industries including aerospace, marine, and biomedical. As a result, it has been the subject of much AM and EBPBF research [6]. An appropriate processing window and scanning strategy for EBPBF Ti-6Al-4V, in order to avoid possible defects such as porosity, swelling, and un-melted powder particles, has been determined by multiple researchers [7,8,9]. Additionally, the influence of process parameters within this window, build orientation, and post-build heat treatments on the resulting microstructure and mechanical properties has been extensively investigated [10,11,12,13,14,15].

These materials may be considered in applications where fracture or impact toughness is a key property, such as in gas turbine fan blades, where foreign or direct object damage may occur. As such, Charpy impact testing can offer a fast and qualitative approach in down-selecting the final parameters for the manufacturing process, after which more complex fracture toughness testing would be recommended to determine the plane strain fracture toughness (K_IC_). The impact energy of EBPBF-produced Ti-6Al-4V has been previously studied, examining the influence of build orientation, powder oxidation, porosity, post-processing, and texture [16,17]. This research expands on these studies through testing a series of five EBPBF process parameter sets across vertical and horizontal build orientations, to determine relationships between process parameters, microstructural characteristics, and impact energy.

## 2. Materials and Methods

### 2.1. Materials

The EBPBF material used in this study was produced from gas-atomised Ti-6Al-4V grade 5 powder feedstock supplied by ARCAM AB™. All specimens were manufactured at the University of Sheffield using an ARCAM system configured with Control 3.2 Service Pack 2 software (Stockholm, Sweden), using virgin powder with a particle size distribution of 45 to 150 µm, where normal distribution values for D_10_, D_50_, and D_90_ were 55.6, 80.7, and 105.7 µm, respectively. Standard process conditions defined by ARCAM for Ti-6Al-4V were used, including initial powder bed temperature, *T*_0_ equal to 923 K, and applied helium shielding.

Oversized 10 mm × 10 mm × 55 mm cuboidal-shaped specimens were built under five different process parameter sets in both the vertical and horizontal orientations, with four specimens of each combination providing a total of 40 specimens. The process parameters investigated are given in Table 1, where *q* is the beam power, *v* is the beam velocity, *h* is the hatch spacing, and *l* is the layer height. Normalised equivalent energy density (*E*_0_*) values were derived from normalised process parameters (q*, v*, l* and h*) based on the research of Thomas et al. [18], with the same thermo-physical properties used to determine the dimensionless parameters.

**Figure 1:**
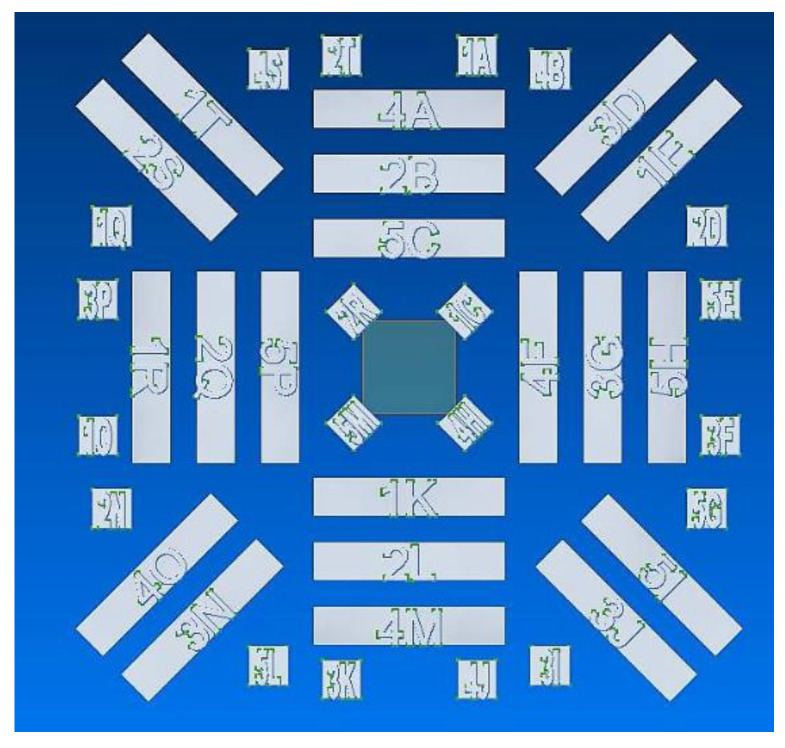
Plan view of specimen distribution on EBPBF build plate.

Figure 1 shows the distribution of specimens across the EBPBF build plate in plan view. Parameter and build orientation differences were spread across the different build plate regions; as such, the influence of build location did not form part of this study. In the diagram, the rectangular-shaped specimens represent the horizontally built test-pieces, whilst the vertically oriented test-pieces are represented by the square-shaped features.

### 2.2. Experimental Methods

**Figure 2:**
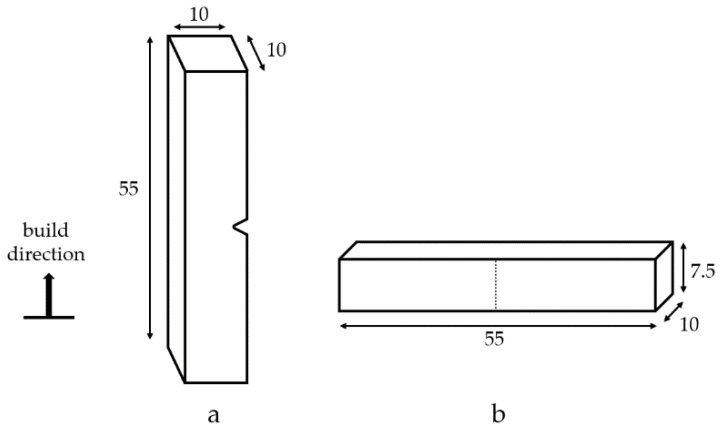
Charpy V-notch specimen dimensions and notch locations relative to build orientation: (**a**) standard size vertically built specimens and (**b**) subsize horizontally built specimens; all dimensions in mm.

Charpy V-notch specimens were machined from the EBPBF cuboids, with the dimensions and notch locations relative to the build orientation shown in Figure 2. Due to a small level of shrinkage in the horizontally built cuboids, which took the final dimension in the build direction as just below 10 mm, these were manufactured to subsize dimensions of 55 mm × 10 mm × 7.5 mm (Figure 2b), with the vertical-built specimens being standard sized at 55 mm × 10 mm × 10 mm (Figure 2a). All specimens contained a 2 mm deep 45° V notch with a 0.25 mm root radius at the centre. All tests were performed on an Instron^®^ MPX (MA, USA) impact test system at room temperature in accordance with ASTM-E23 [19]. Microstructure and fractographic investigations were performed on a Reichert Jung MeF3 (Wetzlar, Germany) optical microscope and a Zeiss EvoLS25 (Oberkochen, Germany) scanning electron microscope (SEM). Specimens were prepared using a standard polishing technique and etched using Kroll’s reagent (2% hydrofluoric acid). In addition, a series of five tests were performed on cast Ti-6Al-4V using the standard specimen size to act as a baseline comparison.

## 3. Results and Discussion

### 3.1. Mechanical Properties

Table 2 provides an overview of the results and standard deviations of the impact tests on the EBPBF materials. The table provides average values taken from four results for each of the five parameter sets, in each of the two different EBPBF build orientations (five for the cast variant). The one exception is for vertical build parameter set 2, where the values were taken from two specimens due to void tests. Energy absorbed was taken directly from each test, and impact energy was calculated by dividing the energy absorbed by the cross-sectional area of the unnotched ligament: 0.8 and 0.6 cm^2^ for the vertical and horizontal specimens, respectively. Converting to specific energy enabled comparisons to be made between the standard and subsize geometries; various studies have been conducted investigating size effects in Charpy testing [20,21]. The range of impact energy values determined here (36–75 J∙cm^−2^) is in line with previous research [16,22].

For all process parameter sets with a beam velocity less than 500 mm∙s^−1^ (parameter sets 2 to 5), the impact energy of the EBPBF materials is greater than the Cast Ti-6Al-4V baseline. There is one exception in the case of horizontal parameter set 4, which has the largest hatch spacing (0.24 mm), thus leading to a very fine microstructure, leading to the production of an impact energy value slightly below the Cast baseline.

Anisotropic impact performance is clear, with vertically oriented specimens consistently exhibiting a higher impact energy than the horizontal specimens across all parameter sets, except parameter set 1, which has the highest beam velocity (778.6 mm∙s^−1^); the impact energy results for both orientations are within 2% of one another. For parameter sets 2 to 5, the average increase in impact energy is 34% from horizontal to vertical orientations. This difference is attributed to the fracture path being predominantly intergranular in nature (see Section 3.3). Therefore, in the vertical specimens, where the columnar prior β grains in the build direction are perpendicular to the crack plane, the crack front has farther to travel (or must pass through the prior β grains), unlike the horizontal specimens, which have a more equiaxed grain structure perpendicular to the crack plane [17].

**Figure 3:**
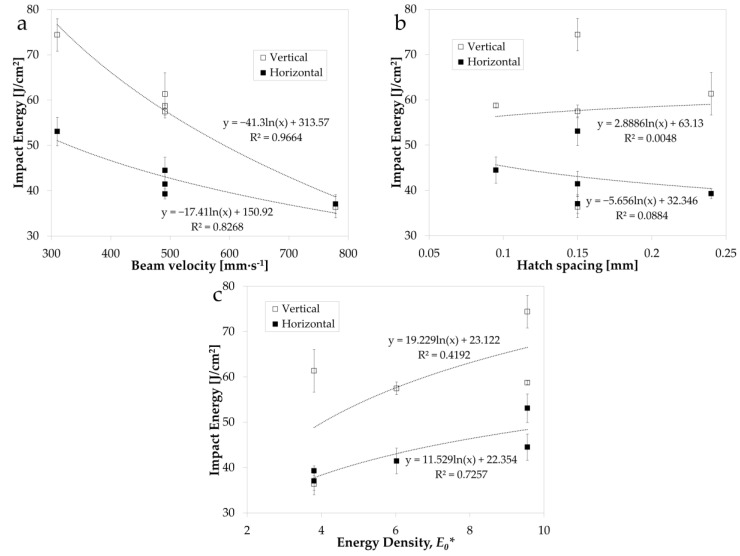
Impact energy in the vertical and horizontal build orientation vs. (**a**) beam velocity, (**b**) hatch spacing, and (**c**) normalised energy density, *E*_0_*.

Figure 3 shows the relationship between impact energy and the varied process parameters of beam velocity (Figure 3a), hatch spacing (the distance between two consecutive laser beam passes) (Figure 3b), and normalised energy density, *E*_0_* (Figure 3c), for both the vertical and horizontal build orientations. A logarithmic trendline was fit to all process–microstructure–property plots presented in this study as a means of assessing the significance of the interdependent relationships. The most prominent process parameter relationship with impact energy is shown to be beam velocity (Figure 3a), with a decrease in impact energy observed with an increase in beam velocity. This relationship then appears to plateau as beam velocity is increased further, with the same relationship revealed for both build orientations. Hatch spacing is revealed to have no clear relationship with impact energy in the process window studied in this research (R^2^ < 0.1). As expected, based on the beam velocity and hatch spacing trends, the normalised energy density, *E*_0_*, exhibits an overall positive trend with impact energy.

### 3.2. Microstructure

**Figure 4:**
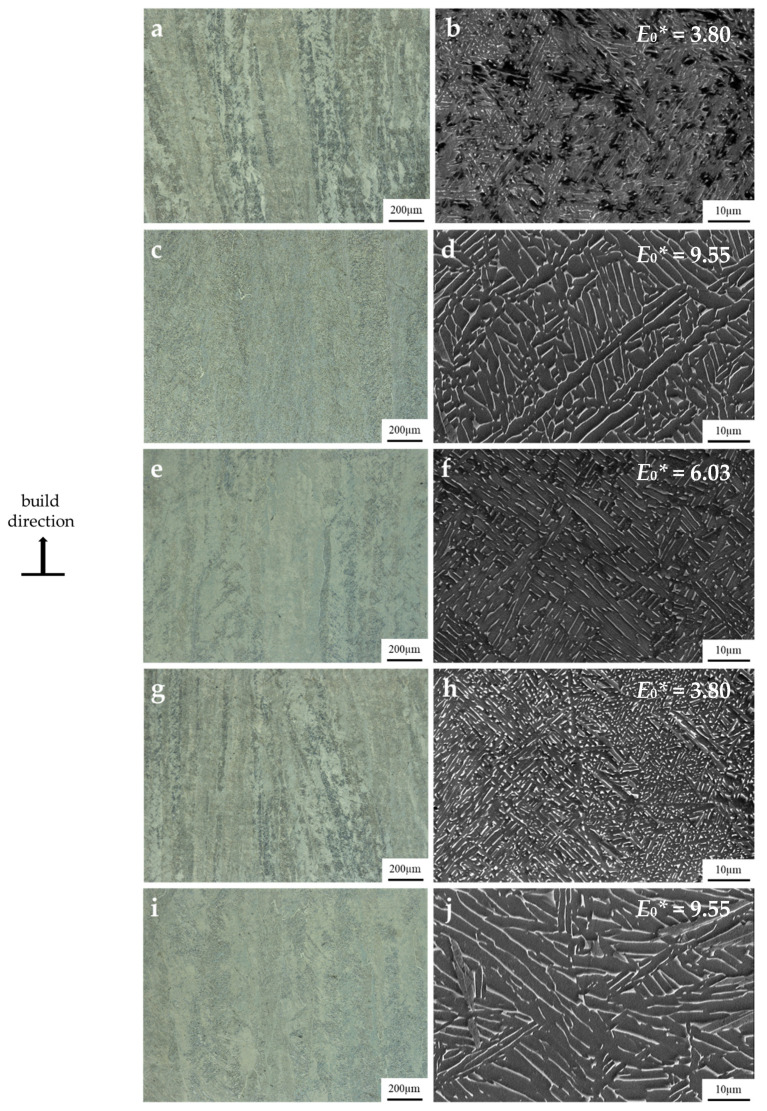
Microstructures of EBPBF specimens in the build plane direction for (**a**,**b**) parameter set 1, (**c**,**d**) parameter set 2, (**e**,**f**) parameter set 3, (**g**,**h**) parameter set 4, and (**i**,**j**) parameter set 5.

The microstructures of the EBPBF parameter sets at low and high magnifications are shown in Figure 4. In each case, the microstructures are taken from the build direction plane as indicated. Across all parameter sets, the bulk microstructure in the build direction consists of columnar prior β grains, with grain boundary α forming on the prior β grain boundaries (Figure 4—low magnification). The formation of these epitaxial prior β grains is due to the successive melting of layers during the EBPBF process and the resulting thermal gradients in the build direction. Within these columnar prior β grains, a typical α + β microstructure is observed with Widmanstätten α platelet and colony morphologies (Figure 4—high magnification). The observed microstructures are consistent with those found in the literature [4,6,23].

The quantitative average microstructural characteristics of prior β grain area, prior β grain width, and α lath width were determined using an intercept method analysing five micrographs for each parameter set. For the α lath width measurements, a minimum of 100 measurements were taken from each 1500× micrograph to ensure statistical significance.

**Figure 5:**
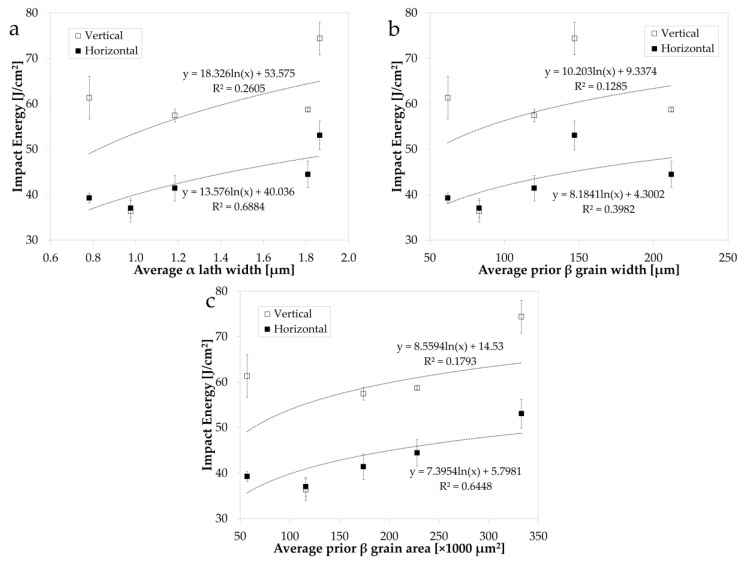
Microstructural characteristics in the build direction of average α lath width, prior β grain width, and prior β grain area vs. processing parameters (**a**) beam velocity, (**b**) hatch spacing, and (**c**) normalised energy density, *E*_0_*.

Figure 5 shows the relationship between the measured microstructural characteristics (α lath width, prior β grain width, and prior β grain area) and the varied process parameters of beam velocity (Figure 5a), hatch spacing (Figure 5b), and normalised energy density, *E*_0_* (Figure 5c). Hatch spacing, as expected, is most strongly related to prior β grain width, with a decreasing hatch spacing resulting in an increase in prior β grain width. While the general relationships of the measured microstructural characteristics with beam velocity and hatch spacing are clear in trend direction, decreasing as both are increased, there was considerable scatter observed. However, through analysing the parameter sets that have a consistent hatch spacing and varied beam velocity (parameter sets 1, 3, and 5), or a consistent beam velocity and varied hatch spacing (parameter sets 2, 3, and 4), these relationships become explicitly defined in each case. This emphasises the importance of considering the normalised energy density, *E*_0_*, of the EBPBF process, which illustrates the most significant relationship with all the microstructural characteristics (R^2^ > 0.8 in each case). This indicates the benefit of combining process parameters to understand the resulting microstructure, combining the influence of the beam velocity and hatch spacing.

**Figure 6:**
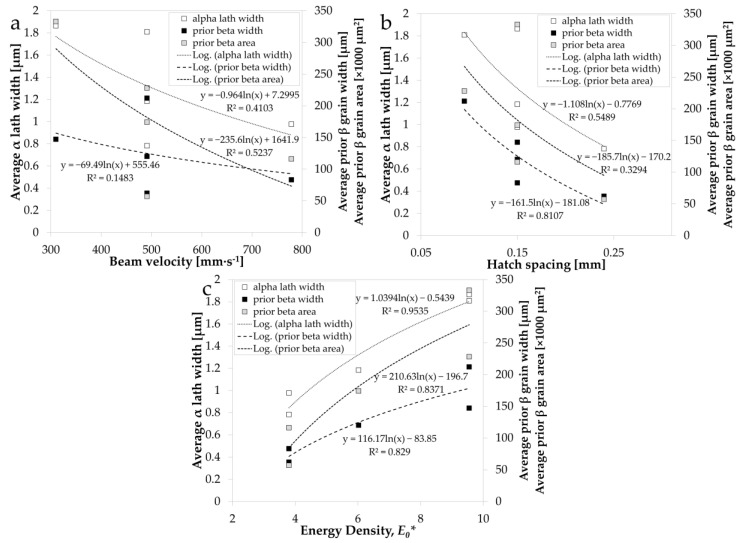
Impact energy in the vertical and horizontal build orientation vs. microstructure characteristics of average (**a**) α lath width, (**b**) prior β grain width, and (**c**) prior β grain area.

Figure 6 shows the relationship between impact energy and the microstructural characteristics, α lath width (Figure 6a), prior β grain width (Figure 6b), and prior β grain area (Figure 6c), of the varied process parameter sets. In each case, a positive trend is observed: as impact energy increases, the average microstructural measures increase concurrently. This corroborates the notion that the favoured fracture path is predominantly intergranular, so a decrease in grain boundary density leads to an increase in impact energy. However, these trends are more prominent in the horizontally oriented specimens than in the vertical specimens, attributed to the relatively low spread of data in the horizontal impact energy results, and to the fact that microstructural analysis was conducted on the build direction plane.

### 3.3. Fractography

**Figure 7:**
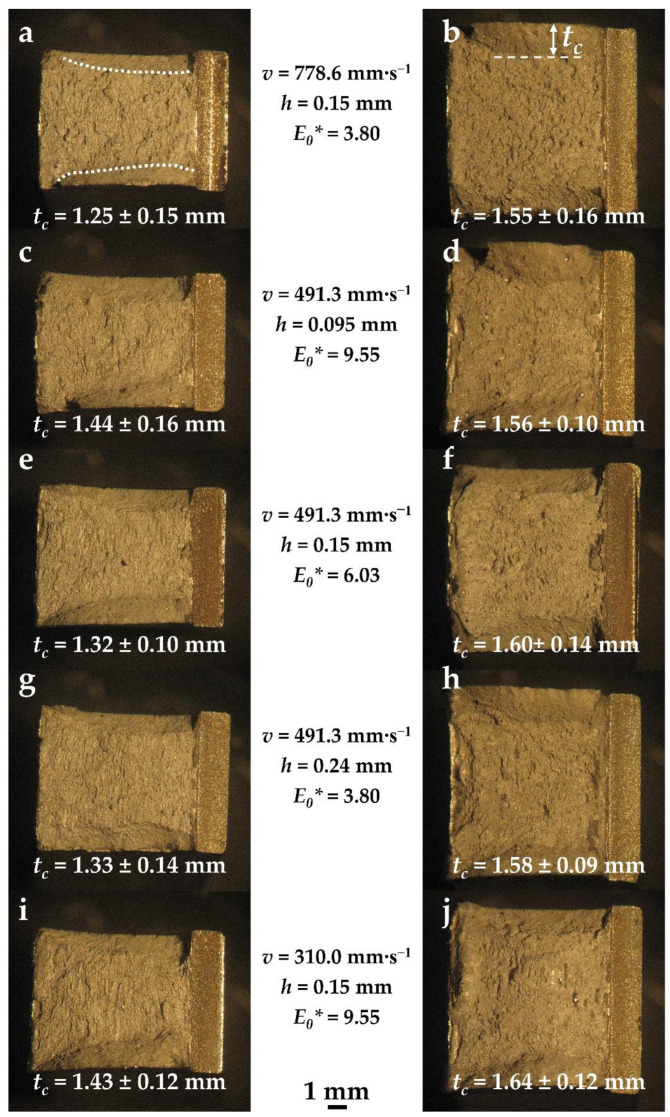
Fracture surfaces of EBPBF Charpy test specimens, with the process parameters indicated; (**a**,**c**,**e**,**g**,**i**) horizontally built specimens are shown on the left and (**b**,**d**,**f**,**h**,**j**) vertically built specimens on the right. Average and standard deviations of the shear lip width (*t_c_*) measurements taken across all tests for each parameter set and build orientation are provided.

Figure 7 presents the fracture surfaces of the Charpy specimens for each of the EBPBF parameter sets and the two build orientations. The horizontally built specimen fractures are shown on the left (Figure 7a,c,e,g,i) and the vertically built specimen fractures are shown on the right (Figure 7b,d,f,h,j), with the process parameter conditions stated. All fracture surfaces reveal significant and relatively symmetric shear lip regions, highlighted in Figure 7a. The width of these shear lip regions, *t_c_*, (Figure 7b) was measured for all specimens, with the average and standard deviation values also provided in Figure 7.

**Figure 8:**
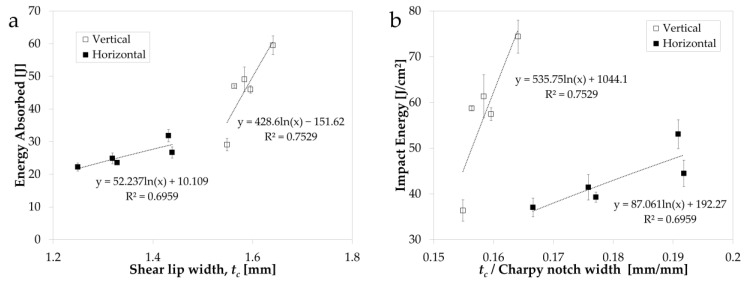
(**a**) Energy absorbed vs. shear lip width (*t*_c_) and (**b**) impact energy vs. ratio of shear lip width (*t_c_*) to Charpy notch width for the vertical and horizontal build orientations.

Figure 8a shows the relationship between the energy absorbed and *t_c_* for the different process parameter conditions and specimen orientations. For both orientations, a positive relationship is revealed overall, with the energy absorbed increasing with increasing *t_c_*. The gradient of the trend for the vertically oriented specimens is almost an order of magnitude greater than that of the horizontally oriented specimens. Furthermore, the average *t_c_* measurements for the vertical specimens are on average 16% greater than the average *t_c_* measurements for the horizontal specimens.

However, to enable a fairer comparison of the *t_c_* measurements between the two build orientations, it was necessary to normalise the values due to the different test specimen sizes used in this research (7.5 and 10 mm for the horizontal and vertical specimens, respectively). Figure 8b shows the relationship between the impact energy and the ratio of *t_c_* to the Charpy notch width. This shows that the shear lip region in the horizontally oriented specimens takes up a larger proportion of the overall fracture surface in comparison to the vertically oriented specimens across all process parameter sets.

**Figure 9:**
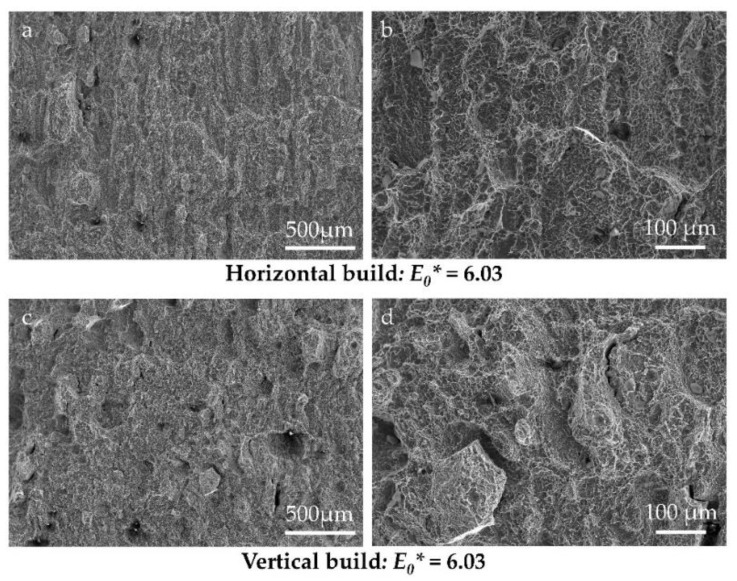
SEM images of Charpy test fracture surfaces taken from specimens built with process parameter set 3 (*E*_0_* = 6.03), (**a**,**b**) horizontal and (**c**,**d**) vertical. Notch is located to the right of the images.

Figure 9 shows SEM images of the fracture surfaces of the horizontally (Figure 9a,b) and vertically (Figure 9c,d) oriented specimens, looking at specimens built with process parameter set 3, which has a normalised energy density *E*_0_* of 6.03. The epitaxial character of EBPBF Ti-6Al-4V is apparent, along with evidence of the intergranular fracture morphologies. The intergranular fracture mechanism, epitaxial character, and microstructural measurements are the key aspects that contribute to the anisotropic impact energy properties presented, as well as the differences revealed between the investigated process parameter conditions.

## 4. Conclusions

- A series of five EBPBF Ti-6Al-4V process parameter sets were investigated in terms of their resulting microstructure and impact energy across the vertical and horizontal build orientations.
- Vertically oriented specimens revealed an average increase in impact energy of 34% compared to the horizontally oriented specimens when beam velocity was less than 500 mm∙s^−1^, attributed to the epitaxial grain structure and its alignment to the Charpy test crack plane.
- Beam velocity was the parameter shown to have the most significant influence on impact energy, which reduced by 69% and 36% for the vertical and horizontal oriented specimens, respectively, when beam velocity was increased from 310.0 to 778.6 mm∙s^−1^.
- Microstructural characteristics of α lath width, prior β grain width, and prior β grain area were determined and related to process parameters and impact energy.

## Figures and Tables

**Table 1:** EBPBF process parameter sets.

Parameter Set	*q*	*v*	*h*	*l*	*E*_0_*
	(W)	(mm∙s^−1^)	(mm)	(mm)	(J∙mm^−2^)
1	300	778.6	0.15	0.07	3.80
2	300	491.3	0.095	0.07	9.55
3	300	491.3	0.15	0.07	6.03
4	300	491.3	0.24	0.07	3.80
5	300	310.0	0.15	0.07	9.55

**Table 2:** Charpy test results on Ti-6Al-4V specimens. Average and standard deviation values are taken from four test specimens in each case except where indicated.

Build Orientation	Parameter Set	Energy Absorbed	SD	Impact Energy	SD
		(J)		(J∙cm^−2^)	
Vertical EBPBF	1	29.1	1.9	36.4	2.4
	2 *	47.0	0.3	58.7	0.4
	3	46.0	1.1	57.5	1.4
	4	49.1	3.8	61.3	4.7
	5	59.5	2.9	74.4	3.6
Horizontal EBPBF	1	22.2	1.2	37.0	2.1
	2	26.7	1.7	44.5	2.9
	3	24.9	1.7	41.4	2.8
	4	23.6	0.7	39.3	1.1
	5	31.8	1.9	53.1	3.2
Cast **		34.1	3.8	42.7	4.8

* based on two specimens; ** based on five specimens.

## Data Availability

The data presented in this study are not publicly available due to forming part of an ongoing study but may be available on request.
